# The effect of ecosystem biodiversity on virus genetic diversity depends on virus species: A study of chiltepin-infecting begomoviruses in Mexico

**DOI:** 10.1093/ve/vev004

**Published:** 2015-06-01

**Authors:** Manuel Rodelo-Urrego, Fernando García-Arenal, Israel Pagán

**Affiliations:** Centro de Biotecnología y Genómica de Plantas (UPM-INIA) and Dpto. de Biotecnología, Campus Montegancedo, Universidad Politécnica de Madrid, Autopista M40 (Km. 38), 28223, Pozuelo de Alarcón, Madrid, Spain

**Keywords:** begomoviruses, *Capsicum annuum glabriusculum*, biodiversity, population genetic diversity, plant–virus interactions, recombination

## Abstract

Current declines in biodiversity put at risk ecosystem services that are fundamental for human welfare. Increasing evidence indicates that one such service is the ability to reduce virus emergence. It has been proposed that the reduction of virus emergence occurs at two levels: through a reduction of virus prevalence/transmission and, as a result of these epidemiological changes, through a limitation of virus genetic diversity. Although the former mechanism has been studied in a few host-virus interactions, very little is known about the association between ecosystem biodiversity and virus genetic diversity. To address this subject, we estimated genetic diversity, synonymous and non-synonymous nucleotide substitution rates, selection pressures, and frequency of recombinants and re-assortants in populations of *Pepper golden mosaic virus* (PepGMV) and *Pepper huasteco yellow vein virus* (PHYVV) that infect chiltepin plants in Mexico. We then analyzed how these parameters varied according to the level of habitat anthropization, which is the major cause of biodiversity loss. Our results indicated that genetic diversity of PepGMV (but not of PHYVV) populations increased with the loss of biodiversity at higher levels of habitat anthropization. This was mostly the consequence of higher rates of synonymous nucleotide substitutions, rather than of adaptive selection. The frequency of recombinants and re-assortants was higher in PepGMV populations infecting wild chiltepin than in those infecting cultivated ones, suggesting that genetic exchange is not the main mechanism for generating genetic diversity in PepGMV populations. These findings provide evidence that biodiversity may modulate the genetic diversity of plant viruses, but it may differentially affect even two closely related viruses. Our analyses may contribute to understanding the factors involved in virus emergence.

## 1 Introduction

One of the defining characteristics of viruses is their high capacity to generate genetic diversity (Holmes 2009). This capacity is directly linked to the continuous appearance of drug-resistant strains and of new viruses that overcome the host immune system or the genetic resistance of crops (García-Arenal and McDonald 2003; Holmes 2009; Pennings 2012; Elena et al. 2014). Indeed, around 150 viral diseases have emerged in humans, plants, and wildlife in the last 60 years at accelerating rates (Dobson and Foufopoulos 2001; Anderson et al. 2004; Jones et al. 2008). A fundamental step to reduce the number of emergent virus diseases, and a long-standing goal for evolutionary virologists, is to understand which factors modulate the genetic diversity of virus populations. Because, by definition, infectious diseases require the interaction between various species, at least the host and the parasite, ecosystem biodiversity may be one of such factors. Indeed, theoretical elaborations hypothesize that biodiversity may determine the genetic diversity of parasite populations, including viruses, through changes in the parasite’s epidemiology. According to this hypothesis, the loss of biodiversity would increase host abundance and density, which in turn would lead to higher transmission rates and a higher prevalence of parasites (Keesing, Holt, and Ostfeld 2006; Burdon and Thrall 2008; Keesing et al. 2010; Ostfeld and Keesing 2012). As a consequence of these epidemiological changes, parasite populations would increase in size, accelerating their evolutionary rates (Scholle et al. 2013; Lanfear, Kokko, and Eyre-Walker 2014), which could ultimately lead to higher genetic diversity.

In the past decade, increasing evidence has linked the loss of ecosystem biodiversity to higher transmission rates and prevalence of viruses and other parasites (reviewed in Keesing et al. 2010; Ostfeld and Keesing 2012; Alexander et al. 2014). However, our understanding on how the loss of biodiversity modulates the genetic diversity of virus populations is scarce and relies mostly on indirect evidence (Holmes 2009; Scholle et al. 2013; Lanfear, Kokko, and Eyre-Walker 2014). This is especially so in the case of plant viruses (Alexander et al. 2014), for which most of the existing evidence comes from analyses of plant-virus interactions under different levels of ecosystem anthropization, which is also the major cause of biodiversity loss (Jones 2009; Keesing et al. 2010). At odds with the hypothesis above, comparative genomics analyses of geminivirus species showed a reduction of virus genetic diversity and of recombination rates with increased ecosystem anthropization (Silva et al. 2011, 2012; Lima et al. 2013). On the other hand, inferences of divergence times for several groups of plant viruses have traced their radiation to the origin or the intensification of agriculture (Fargette et al. 2008; Duffy and Holmes 2008; Gibbs et al. 2008; Pagán and Holmes 2010, but see Lefeuvre et al. 2010; Yasaka et al. 2014). These works proposed that such bursts of genetic diversity could have co-occurred with the intrinsic loss of biodiversity characteristic of agroecosystems. Altogether, these results suggest that the effect of ecosystem anthropization, and the concurrent biodiversity loss, on the genetic diversity of plant viruses may depend on the relative importance of mutation and recombination. These apparently contradictory results derive from analyses that combine viral sequences obtained from different hosts, and from different geographic locations. Hence, it is difficult to disentangle whether the reported changes in plant virus genetic diversity are indeed due to ecosystem biodiversity or the result of host and/or local adaptation processes. As a consequence, it is still unclear whether and how habitat anthropization and biodiversity loss affects the genetic diversity of plant virus populations.

In this work, we analyzed the effect of ecosystem biodiversity on the genetic diversity of virus populations using sequence data of two plant begomovirus species that infect wild pepper or ‘chiltepin’, *Capsicum annuum* var. *glabriusculum* (Dunal) Heiser and Pickersgill, in Mexico. To avoid the limitations of previous works, virus isolates were collected from populations of the same host (chiltepin), within the natural distribution area of the host in Mexico, and from habitats under three levels of anthropization: populations where human intervention is limited to occasional harvesting of chiltepin fruits were considered as wild (Votava, Nabham, and Bosland 2002). Chiltepin populations either tolerated or protected within anthropic habitats were considered as an intermediate level of anthropization (i.e., ‘let-standing’ plants *sensu* Casas et al. (2007)). Cultivated populations, where chiltepin is under incipient domestication, were considered as the highest level of anthropization. Importantly, we have recently reported that increasing level of anthropization of chiltepin populations is associated with lower biodiversity (plant species richness of the habitat and host species genetic diversity) and with higher host plant density. Also, we showed that biodiversity is the main predictor of virus infection risk (Pagán et al. 2012). Hence, the currently coexisting three levels of anthropization and the association between level of anthropization, biodiversity, and risk of virus infection make chiltepin a unique host to analyze the effect of biodiversity in the genetic diversity of plant virus populations.

In the Mexican chiltepin populations, the most prevalent plant viruses are two species of the genus *Begomovirus* (Family *Geminiviridae*): *Pepper golden mosaic virus* (PepGMV) and *Pepper huasteco yellow vein virus* (PHYVV) (Rodelo-Urrego et al. 2013). Both viruses have a two-segmented genome (DNA-A and DNA-B) of circular single-stranded DNA. DNA-A encodes viral proteins required for replication, control of gene expression, overcoming of host defenses, and encapsidation (replication protein, Rep; replication enhancer, REn; transcriptional activator protein, TrAP; coat protein, CP), whereas DNA-B encodes two proteins involved in intra- and inter-cellular movement (nuclear shuttle protein, NSP; movement protein, MP) (Torres-Pacheco et al. 1993, 1996). PepGMV and PHYVV are horizontally transmitted by the whitefly *Bemisia tabaci* Gennadius (Homoptera, Aleyrodidae) in a persistent manner and are not seed transmitted (Rojas et al. 2005; Navas-Castillo, Fiallo-Olive, and Sanchez-Campos 2011). These viruses were first described in Mexico in the early 1990s infecting pepper crops, and available data strongly suggest that they have a narrow natural host range, as they have been reported infecting only *Capsicum* and some related genera within the *Solanaceae* (Brown and Poulos 1990; Garzón-Tiznado et al. 1993; Torres-Pacheco et al. 1996; Navas-Castillo, Fiallo-Olive, and Sanchez-Campos 2011). We have previously shown that the higher PepGMV and PHYVV prevalence in chiltepin populations at increasing levels of habitat anthropization is explained by biodiversity loss (Pagán et al. 2012; Rodelo-Urrego et al. 2013). Our data also suggested that higher virus prevalence could be associated with increased transmission rates, as virus infection was more frequent in environmental conditions that favored the presence of the vector *B. tabaci* (Rodelo-Urrego et al. 2013). This observation is compatible with higher prevalence and transmission rate at decreasing biodiversity, i.e., those epidemiological changes predicted to affect virus genetic diversity, occurring in chiltepin-infecting PepGMV and PHYVV populations.

We obtained the full-length sequences of eighty-nine PepGMV and PHYVV isolates from host populations with different levels of biodiversity/ anthropization. We estimated the synonymous and non-synonymous substitution rates and the selection pressures in the corresponding virus populations. Because both viruses belong to the family *Geminiviridae*, for which recombination is thought to play a key role in generating genetic diversity, we also determined the frequency of recombinants (and re-assortment of genomic segments) rates in PepGMV and PHYVV populations. Using these data, we explored the role of biodiversity, plant density, and virus prevalence in the intra-specific genetic diversity of virus populations. Our results indicate that genetic diversity of PepGMV (but not of PHYVV) populations increases with the loss of biodiversity at higher levels of habitat anthropization. This is primarily due to a higher rate of synonymous nucleotide substitutions, rather than to mutations that lead to amino acid changes. Interestingly, the frequency of recombinants and re-assortants was higher in PepGMV populations infecting wild chiltepin than in those infecting cultivated ones, which suggests that genetic exchange plays a secondary role in generating genetic diversity of this virus. Hence, our results provide evidence that biodiversity may be relevant in determining the genetic diversity of plant viruses, as is the case of PepGMV, but that this finding cannot be extended even to closely related viruses such as PHYVV.

## 2 Materials and methods

### 2.1 Collection of PepGMV and PHYVV isolates and sequencing of full-length genomic DNAs

Field surveys are described in Pagán et al. (2012), and detection of PepGMV- and PHYVV-infected plants was as described in Rodelo-Urrego et al. (2013). Data on species richness, chiltepin genetic diversity, and plant density, and PepGMV and PHYVV prevalence in the surveyed chiltepin populations are available in Pagán et al. (2012) and Rodelo-Urrego et al. (2013). Plants positive for PepGMV and PHYVV infection were enriched in circular DNAs with TempliPhi® kit (GE Healthcare Life Sciences, Chalfont St Giles, UK), following manufacturer’s protocol. Utilizing the product of this reaction as template, the complete nucleotide sequence of both genomic DNAs was obtained using different sets of primers. Primers were designed to produce five fragments in such a way that adjacent fragments overlapped in at least 120 nt. Primer sequences and their locations in the genome of a reference strain are summarized in Supplementary Table S1. Overlapping regions of adjacent fragments presented 99–100 per cent nucleotide identity. Thus, the complete nucleotide sequence of each genomic DNA was determined by assembling the five fragments using Lasergene’s SeqMan Pro (DNASTAR, Madison, WI). To avoid artifactual recombination profiles and sequencing of low frequency variants non-representative of the virus population, no cloning step was performed prior to genome sequencing.

**Figure 1.**
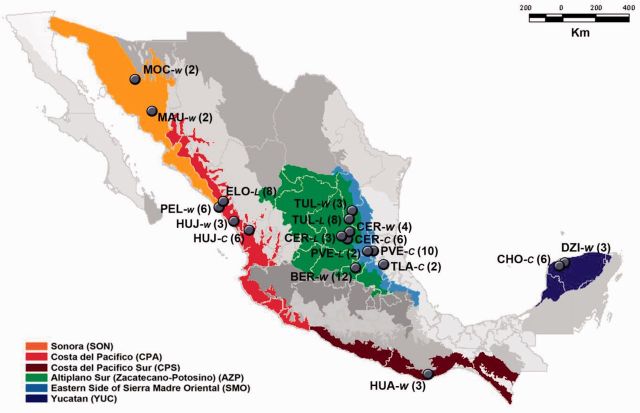
Location of sampled chiltepin populations. Map shows the location of populations from wild (W), let-standing (L), and cultivated (C) populations within six biogeographical provinces in Mexico. Number of PepGMV and PHYVV sequences from each location is shown between parentheses. Full names of each location can be found in Supplementary Table S2.

Sequencing resulted in two datasets containing the complete genomic sequence of forty-seven PepGMV isolates (nineteen from wild, ten from let-standing, and eighteen from cultivated habitats) and forty-two PHYVV isolates (nineteen from wild, eleven from let-standing, and twelve from cultivated habitats). Sequences were aligned beginning at the nicking site in the invariant non-anucleotide located at the origin of replication. In all cases, alignments were constructed using MUSCLE 3.7 (Edgar 2004) and adjusted manually according to the amino acid sequences using Se-Al (Rambaut 1996). The locations and years of sampling are presented in Fig. 1 and Supplementary Table S2.

### 2.2 Association analyses

Association between habitat and PepGMV and PHYVV phylogenies was assessed using Parsimony Score, Association Index, and Monophyletic Clade Size (MC) statistics (Parker, Rambaut, and Pybus 2008). Parsimony Score and Association Index statistics indicate the phylogenetic clustering of viruses that come from the same habitat across entire trees. The MC statistic assesses the association between habitat and virus phylogeny by estimating the size of the largest cluster of sequences coming from the same habitat. These analyses were performed using BaTS (Parker, Rambaut, and Pybus 2008). Real-tree distributions were obtained using BEAST v1.7.5 (Drummond and Rambaut 2007). Null distributions for the three statistics were obtained from 1,000 data replications.

### 2.3 Estimation of genetic and haplotype diversity, and of selection pressures

Genetic diversity (*π*) was estimated for each genomic DNA molecule and for each gene, as average pairwise nucleotide differences, using the Tamura-Nei nucleotide substitution model as implemented in MEGA 5.2.2 (Tamura et al. 2011). Standard errors (SE) of each measure were based on 1,000 replicates bootstrap. Haplotype diversity indexes were calculated in DnaSP v.5 (Rozas et al. 2003).

Selection pressures for each gene were measured as the averaged site-specific ratio between the mean number of non-synonymous (*d*_N_) and synonymous (*d*_S_) nucleotide substitutions per site (*d*_N_/*d*_S_) using the single-likelihood ancestor counting, the fixed effect likelihood, and the random effects likelihood methods implemented in the HyPhy package (Kosakovsky-Pond and Frost 2005). Because the three methods led to the same conclusions, only the single-likelihood ancestor counting results are shown. In all cases, *d*_N_/*d*_S_ estimates were based on input neighbor-joining trees inferred using the MG94 nucleotide substitution model. Individual values of *d*_N_ and *d*_S_ were also obtained. To test for the robustness of these estimates against the effects of recombination, we estimated selection pressures in sequence datasets where (1) recombinant sequences and (2) recombinant fragments were removed. Analyses in these two types of sequence datasets led to similar conclusions than using all the sequences.

Because the number of sequences used to obtain estimates of genetic diversity and selection pressures was generally small, we tested the robustness of our estimates against effect of a single sequence performing a jackknife analysis. Since real and jackknifed estimates did not significantly differ in any case, for simplicity only estimates of real datasets are presented in the text.

### 2.4 Detection of recombination and pseudorecombination

We determined the occurrence of recombination within and between PepGMV and PHYVV populations for each genomic DNA molecule. To do so, we utilized forty-seven PepGMV and forty-two PHYVV fully sequenced isolates. Recombination breakpoints were detected using four methods, based on different assumptions (Posada 2002), and available in RDP4 (http://darwin.uvigo.es/rdp/rdp.html): RDP, BOOTSCAN/RECSCAN, Siscan, and Chimaera, with default parameters (Martin et al. 2010). Statistical significance was obtained using a Bonferroni-corrected cutoff of *P* ≤ 0.05. To minimize false positives, only recombination signals detected by the four methods (*P* < 0.05) were accounted as true events (RDP project files are available upon request). Two recombinant sequences were considered to belong to the same recombination profile when: (1) they shared the same major and minor parentals and (2) a recombination breakpoint in one sequence was <50 nt apart (window size in the recombination detection analysis) of the equivalent recombination breakpoint in the other sequence. The frequency of recombination profiles was used for statistical analyses.

The frequency of pseudorecombination in PepGMV and PHYVV populations was explored by analyzing the topological congruence between the maximum likelihood phylogenies of DNA-A and DNA-B of each virus. We used the CopyCat program (Meier-Kolthoff et al. 2007), which incorporates a wrapper for the program ParaFit (Legendre, Desdevises, and Bazin 2002). ParaFit is a distance-based approach that assesses the fit between DNA-A and DNA-B phylogenetic distance matrices by transforming them into principal coordinates. Statistical significance was evaluated by performing 9,999 permutations to test the null hypothesis of no congruence between DNA-A and DNA-B trees. The same analyses were performed using the program Jane 4 (http://www.cs.hmc.edu/∼hadas/jane/), which uses a topology-based method (Conow et al. 2010), with comparable results (available upon request). Pseudorecombination was also analyzed by concatenating the sequences of the DNA-A and DNA-B of each virus isolate and scanning these sequences for recombination breakpoints near the artificial joint between the two genomic DNAs as described above.

### 2.5 Statistical analyses

Differences in genetic diversity, synonymous and non-synonymous substitution rates, and selection pressures were calculated using parametric (general linear models) and non-parametric (permutation tests) analyses. Since both approaches led to similar conclusions, for simplicity, only general linear model analyses are shown. Variation in the frequency of recombinants between PepGMV and PHYVV, between genomic DNAs, and between levels of anthropization of the host population were analyzed by Fisher’s exact test, with a Yates correction for small sample size when necessary (Sokal and Rohlf 1995). Bivariate tests were used to analyze the association between ecological factors of the chiltepin populations (species diversity as number of plant species, genetic diversity as expected heterozygosity, and plant density as plants/m^2^) and evolutionary parameters of the virus populations (genetic diversity, selection pressures, rate of synonymous and non-synonymous mutations, and frequency of recombinant profiles). Evolutionary parameters used in these bivariate tests were estimated grouping sequences according to population, and eight populations with at least four sequences each were retained for the analysis. Linear and non-linear models were considered in each bivariate analysis, and the model with the highest *r*-value was chosen as the best explaining the relationship between each pair of variables. In addition, for each bivariate analysis model, selection analyses were performed (Burnham and Anderson 2002). Linear and non-linear model were ranked according to Akaike Information criteria (AIC) scores (R library: AICcmodavg), and the model with the lowest AIC was selected as the best explanatory model. In all cases, both approaches led to the same results. Statistical analyses were performed using the software package SPSS 20 (SPSS Inc., Chicago, IL).

## 3 Results

### 3.1 Genetic diversity of chiltepin-infecting PepGMV and PHYVV populations

Overall, haplotype diversity indexes calculated for each DNA component of PepGMV and PHYVV were equal to 1.0, indicating that each isolate had a different haplotype. Genetic diversity (*π*) was significantly lower in DNA-A than in DNA-B for both PepGMV (0.049 ± 0.003 vs. 0.097 ± 0.009; *F*_1,2161_ = 273.1; *P* < 1 × 10^−^^4^) and PHYVV (0.053 ± 0.004 vs. 0.125 ± 0.006; *F*_1,1721_ = 191.7; *P* < 1 × 10^−^^4^). Both viruses showed similar genetic diversity in the DNA-A (*F*_1,1941_ = 1.23; *P* = 0.060), but *π* was lower in the DNA-B of PepGMV than in that of PHYVV (*F*_1,1941_ = 41.92; *P* < 1 × 10^−^^4^) (Table 1). Genetic diversities were also obtained for each gene within the PepGMV and the PHYVV genomes (Supplementary Table S3). In both viruses, genes from DNA-A presented lower genetic diversities than those in DNA-B (*F* > 586.57; *P* < 1 × 10^−^^4^). For PepGMV, all genes encoded by the DNA-A showed similar *π* values (*π*: 0.047–0.050; *F*_3,4323_ = 2.42; *P* = 0.172), whereas in DNA-B, the *MP* gene presented lower genetic diversity than the *NSP* gene (*π*: 0.083 ± 0.008 vs. 0.111 ± 0.009; *F*_1,2161_ = 15.06; *P* < 1 × 10^−^^4^). Similarly, for PHYVV, all genes contained in the DNA-A showed similar *π* values (*π*: 0.046–0.069; *F*_3,3443_ = 1.05; *P* = 0.278); and in the DNA-B, the *MP* had a lower genetic diversity than the NSP gene (*π*: 0.079 ± 0.006 vs. 0.170 ± 0.014; *F*_1,1721_ = 110.0; *P* < 1 × 10^−^^4^) (Supplementary Table S3).

**Table 1.** Genetic diversity (*π*), *d*_N_/*d*_S_, *d*_N_, and *d*_S_ values in the PepGMV and PHYVV genomic DNAs.

Virus	Habitat^a^	*π*^b^		*d*_N_/*d*_S_^c^		*d*_N_^c^		*d*_S_^c^	
		DNA-A	DNA-B	DNA-A	DNA-B	DNA-A	DNA-B	DNA-A	DNA-B
PepGMV	Wild	0.025 ± 0.003	0.091 ± 0.010	0.217 ± 0.018	0.116 ± 0.008	0.017 ± 0.001	0.040 ± 0.002	0.078 ± 0.004	0.281 ± 0.009	
	Let-standing	0.043 ± 0.002	0.098 ± 0.010	0.168 ± 0.012	0.090 ± 0.035	0.018 ± 0.001	0.024 ± 0.002	0.107 ± 0.006	0.270 ± 0.009
	Cultivated	0.068 ± 0.002	0.105 ± 0.008	0.157 ± 0.012	0.112 ± 0.005	0.022 ± 0.001	0.033 ± 0.002	0.140 ± 0.006	0.349 ± 0.013
	All^d^	0.049 ± 0.003	0.097 ± 0.009	0.167 ± 0.004	0.109 ± 0.002	0.021 ± 0.001	0.034 ± 0.001	0.124 ± 0.002	0.316 ± 0.004
PHYVV	Wild	0.059 ± 0.005	0.121 ± 0.006	0.139 ± 0.016	0.107 ± 0.008	0.022 ± 0.001	0.037 ± 0.001	0.159 ± 0.005	0.346 ± 0.011
	Let-standing	0.057 ± 0.004	0.124 ± 0.006	0.129 ± 0.051	0.115 ± 0.005	0.021 ± 0.001	0.039 ± 0.002	0.139 ± 0.012	0.344 ± 0.017
	Cultivated	0.053 ± 0.006	0.129 ± 0.007	0.141 ± 0.008	0.111 ± 0.003	0.020 ± 0.001	0.040 ± 0.002	0.159 ± 0.009	0.363 ± 0.019
	All^d^	0.053 ± 0.004	0.125 ± 0.006	0.135 ± 0.005	0.113 ± 0.002	0.021 ± 0.001	0.039 ± 0.001	0.157 ± 0.002	0.349 ± 0.005

^a^Level of anthropization (wild; let-standing; cultivated; ALL: wild + let-standing + cultivated).

^b^Values are mean ± standard errors based in 1,000 replicates bootstrap.

^c^Values are mean ± standard error based on pairwise determination of *d*_N_/*d*_S_, *d*_N_, and *d*_S_.

^d^Values are mean ± standard error based on values for the four genes of DNA-A concatenated, and on the two genes of DNA-B.

To analyze how anthropization of the host population affected the genetic diversity of PepGMV and PHYVV, we first performed association analyses between habitat and virus phylogenies, which indicated that sequences of PepGMV and PHYVV clustered significantly according to habitat, either wild or cultivated (*P* < 0.038), with the exception of DNA-B sequences of PHYVV from cultivated habitats (*P* = 0.487) (Supplementary Table S4). Hence, PepGMV and PHYVV populations are, in general, genetically structured according to habitat. Thus, we calculated *π* in populations of both viruses belonging to wild, let-standing, and cultivated habitats (Table 1). Genetic diversity in the DNA-A of PepGMV differed among habitats (*F*_2,368_ = 82.66; *P* < 1 × 10^−^^4^). The *π* value was lower in wild than in let-standing populations, and both were lower than in cultivated populations (0.025 ± 0.001, 0.043 ± 0.002, and 0.068 ± 0.002, respectively; *P* < 0.014). Also, *π* values in the DNA-B of PepGMV depended on the habitat (*F*_2,368_ = 7.68; *P* = 1 × 10^−^^3^), being higher in cultivated than in wild habitats (*π*: 0.105 ± 0.008 vs. 0.091 ± 0.010; *P* = 3 × 10^−^^3^) and intermediate in let-standing populations (*π*: 0.098 ± 0.010). For PHYVV, differences according to habitat anthropization were not found either in the DNA-A (*π*: 0.053–0.059; *F*_2,291_ = 1.92; *P* = 0.149) or in the DNA-B (*π*: 0.121–0.129; *F*_2,291_ = 0.96; *P* = 0.386). The same patterns were observed when each gene was analyzed separately (Supplementary Table S3). Thus, higher levels of habitat anthropization were associated with higher genetic diversity of PepGMV but did not affect PHYVV.

### 3.2 Selection pressures in chiltepin-infecting PepGMV and PHYVV populations

We explored whether the observed changes in the genetic diversity of PepGMV populations at increasing habitat anthropization were the consequence of adaptive and/or neutral evolution. To do so, we estimated selection pressures as *d*_N_/*d*_S_ ratio (*ω*), as well as *d*_N_ and *d*_S_ values, for each genomic component and gene of PepGMV isolates in each habitat. The same data were also estimated for PHYVV isolates (Table 1 and Supplementary Table S3).

For PepGMV, average *d*_N_/*d*_S_ in the DNA-A was higher in wild than in let-standing and cultivated populations (*ω*: 0.217 ± 0.018 vs. 0.0168–0.157 ± 0.012; *F*_2,368_ = 13.07; *P* < 1 × 10^−^^4^) (Table 1). Similar results were obtained for the *CP* and *TrAP* genes (*F*_2,368_ > 4.89; *P* < 9 × 10^−^^3^), but *d*_N_/*d*_S_ values of the *REn* and *Rep* genes did not differ across habitats (*F*_2,368_ < 2.52; *P* > 0.114) (Supplementary Table S3). In accordance with these results, the frequency of codons under purifying selection in the four genes of the DNA-A of PepGMV was higher in cultivated than in wild habitats (5 per cent vs. 2 per cent; *χ*^2^_1_ < 7.81, *P* > 0.005), the rest of codons being under neutral evolution. In the DNA-B, *d*_N_/*d*_S_ was similar in the three habitats (*ω*: 0.090–0.112; *F*_2,368_ = 1.15; *P* = 0.319), with the same trend in both the *MP* and the *NSP* genes (*F*_2,368_ < 2.48; *P* > 0.116) (Table 1 and Supplementary Table S3). The frequency of codons under purifying selection did not vary across habitats (14 per cent vs. 18 per cent; *χ*^2^_1_ < 2.32, *P* > 0.128). No codons under positive selection were found. For PHYVV, *d*_N_/*d*_S_ in the DNA-A did not depend on the level of habitat anthropization, either considering all genes together (*ω*: 0.129–0.141; *F*_2,291_ = 0.411; *P* = 0.663) or each gene individually (*F*_2,291_ < 2.26; *P* > 0.106). Selection pressures in the DNA-B also did not differ between habitats (*ω*: 0.107–0.115; *F*_2,291_ = 0.726; *P* = 0.485), and similar results were obtained for the *MP* and the *NSP* genes (*F*_2,291_ < 1.65; *P* > 0.195) (Table 1 and Supplementary Table S3). Accordingly, the frequency of codons under purifying selection did not vary across habitats in neither DNA (2 per cent vs. 3 per cent and 11 per cent vs. 13 per cent, for DNA-A and DNA-B, respectively; *χ*^2^_1_ < 0.54, *P* > 0.462). No codons under positive selection were found in the PHYVV genome.

Average *d*_N_ in the DNA-A of PepGMV was higher in cultivated than let-standing and wild populations (*d*_N_: 0.022 ± 0.001 vs. 0.018–0.017 ± 0.001; *F*_2,368_ = 10.09; *P* < 1 × 10^−^^4^). The same result was obtained for the *REn* and *Rep* genes (*F*_2,368_ > 43.30; *P* < 1 × 10^−^^4^), but *d*_N_ of the *CP* and *TrAP* was not affected by the level of habitat anthropization (*F*_2,368_ > 0.189; *P* < 0.828). In the DNA-B, *d*_N_ was higher in cultivated than in let-standing and wild populations, either considering the complete genomic segment (*d*_N_: 0.040 ± 0.002 vs. 0.024–0.032 ± 0.002; *F*_2,368_ = 12.82; *P* < 1 × 10^−^^4^) or the *MP* and *NSP* genes separately (*F*_2,368_ > 7.85; *P* < 1 × 10^−^^4^) (Table 1 and Supplementary Table S3). For PHYVV, habitat anthropization did not affect *d*_N_ either in the DNA-A (*d*_N_: 0.020–0.022; *F*_2,291_ = 2.72; *P* = 0.067) or in the DNA-B (*d*_N_: 0.039–0.043; *F*_2,291_ = 1.13; *P* = 0.324) (Table 1), and the same was found when each gene was analyzed separately (*F*_2,291_ < 2.20; *P* > 0.060) (Supplementary Table S3).

Average *d*_S_ in the DNA-A of PepGMV was higher in cultivated than in let-standing and wild habitats (*d*_S_: 0.140 ± 0.005 vs. 0.107–0.078 ± 0.006; *F*_2,368_ = 50.79; *P* < 1 × 10^−^^4^) (Table 1). Similar results were obtained when each gene was analyzed separately (*F*_2,368_ > 45.82; *P* < 1 × 10^−^^4^) (Supplementary Table S3). For the DNA-B, *d*_S_ was again higher in cultivated than in let-standing and wild populations (*d*_S_: 0.349 ± 0.013 vs. 0.270–0.281 ± 0.009; *F*_2,368_ = 12.15; *P* < 1 × 10^−^^4^) (Table 1), and the same trend was observed for each DNA-B gene separately (*F*_2,368_ > 12.00; *P* < 1 × 10^−^^4^) (Supplementary Table S3). For PHYVV, *d*_S_ in the DNA-A did not depend on the level of habitat anthropization, either when all genes were considered together (*d*_S_: 0.139–0.159; *F*_2,291_ = 2.07; *P* = 0.128) or each of them separately (*F*_2,291_ < 1.57; *P* > 0.210) (Supplementary Table S3). Similarly, habitat anthropization did not affect *d*_S_ values of the DNA-B considering all genes together (*d*_S_: 0.344–0.363; *F*_2,291_ = 0.37; *P* = 0.695), and the *MP* and *NSP* genes individually (*F*_2,291_ < 1.17; *P* > 0.313) (Table 1 and Supplementary Table S3).

In summary, genes of PepGMV and PHYVV are under purifying selection, which is stronger in the DNA-B. Both *d*_N_ and *d*_S_ are generally smaller in DNA-A than in DNA-B genes, which is compatible with the higher genetic diversity that we observed in the DNA-B of the two studied begomoviruses. Finally, habitat anthropization is associated with a smaller increase of *d*_N_ (adaptive mutations), and with a much larger increase of *d*_S_ (neutral mutations), in the DNA-A and DNA-B of PepGMV. This could explain the observed changes in genetic diversity and selection pressures across habitats.

### 3.3 Frequency of recombinants in chiltepin-infecting PepGMV and PHYVV populations

As recombination is thought to play a key role in generating genetic diversity of begomoviruses (Lefeuvre and Moriones 2015), we analyzed the frequency of recombinants in PepGMV and PHYVV populations and their distribution across levels of anthropization.

The DNA-A and DNA-B full-length sequences of forty-seven PepGMV isolates and of forty-two PHYVV isolates were used to determine the frequency of inter- and intra-specific recombinants in the virus populations. Only six DNA-B sequences were detected as inter-specific recombinants between PepGMV and PHYVV, and the rest of recombinant isolates were intra-specific (Supplementary Figs S1 and S2). For PepGMV, recombination signal was detected in eighteen DNA-A and twenty DNA-B sequences, with 57 per cent (27/47) of isolates being recombinants in at least one of the DNA components (Table 2 and Supplementary Fig. S1). For PHYVV, six recombinant sequences in the DNA-A and nine in the DNA-B were detected, with 31 per cent (13/42) of isolates showing recombination signal in at least one DNA component (Table 2 and Supplementary Fig. S2). For both viruses, frequency of recombinants in the DNA-A was similar to that in the DNA-B of the same virus (*χ*^2^_1_ < 0.73, *P* > 0.55). However, PepGMV showed a higher frequency of recombinants than PHYVV either considering recombinants in at least one DNA component or analyzing recombination in each genomic component separately (*χ*^2^_1_ > 4.51, *P* < 0.04). Recombinant isolates were found in populations under the three levels of human management and in all the years sampled (Supplementary Figs S1 and S2).

**Table 2.** Number of intra-specific recombinants and of breakpoints in the genome of chiltepin-infecting PepGMV and PHYVV isolates.

Virus		All		Wild		Let-standing		Cultivated	
		Recombinants^a^	Breakpoints^b^	Recombinants^a^	Breakpoints^b^	Recombinants^a^	Breakpoints^b^	Recombinants^a^	Breakpoints^b^
PepGMV	DNA-A	38% (18/47)	20	63% (12/19)	16	40% (4/10)	5	11% (2/18)	4
	DNA-B	43% (20/47)	21	42% (8/19)	19	50% (5/10)	14	39% (7/18)	8
	Both^c^	57% (27/47)	41	68% (13/19)	35	70% (7/10)	19	39% (7/18)	12
PHYVV	DNA-A	14% (6/42)	5	5% (1/19)	2	18% (2/11)	4	25% (3/12)	3
	DNA-B	21% (9/42)	19	21% (4/19)	10	18% (2/11)	4	25% (3/12)	6
	Both^c^	31% (13/42)	24	21% (4/19)	12	27% (3/11)	8	50% (6/12)	9

Virus isolates may have more than one recombinant fragment in the same genomic DNA. The same recombination breakpoint may appear in isolates from different habitats.

^a^Percentage of recombinant sequences (number of recombinants out of the total number of isolates sequenced).

^b^Number of different breakpoints in the recombinant sequences.

^c^Number of recombinants in at least one of the genomic DNAs out of the total number of isolates sequenced and the corresponding number of breakpoints.

**Figure 2.**
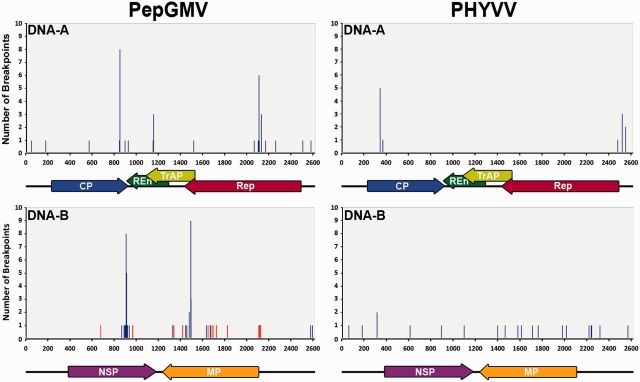
Distribution and abundance of recombination breakpoints in both the DNA-A and the DNA-B of PepGMV and PHYVV. *x* axis indicates the position (nt) of each intra-specific (blue line) and inter-specific (red line) recombination breakpoint in each component of the viral genome. Colored arrows denote the position and orientation of each gene. CP, blue; REn, green; TrAP, yellow; Rep, red; NSP, purple; MP, orange. *y* axis indicates the number of virus isolates in which each recombination breakpoint was detected.

Recombination breakpoints were also mapped. In PepGMV, twenty recombination breakpoints were detected in the DNA-A and twenty-one in the DNA-B, whereas in PHYVV, five and nineteen breakpoints were detected in DNA-A and DNA-B, respectively (Fig. 2). Localization of these recombination breakpoints in the virus genome components indicated the presence of several recombination ‘hotspots’ (Fig. 2). Two ‘hotspots’ in the DNA-B were detected associated with inter-specific recombination: regions between nucleotides 1646–1722 and 2106–2122 (both in the *MP* gene), with 29 per cent (4/14) of the recombination breakpoints each (Fig. 2). All the inter-specific recombinants had a PepGMV isolate as a major parental. Intra-specific recombination ‘hotspots’ were also detected in both PepGMV and PHYVV genomes. In PepGMV, two recombination ‘hotspots’ were found in DNA-A. One between nucleotides 845–923 (3’-end of the *CP* gene), which included 20 per cent (4/20) of recombination breakpoints, with one of these present in eight isolates. The second was located between nucleotides 2066–2169 (central region of the *Rep* gene) and included 30 per cent (6/20) of the breakpoints, with one of them present in six isolates and other present in three (Fig. 2). Also, two recombination ‘hotspots’ were found in the DNA-B: the region between nucleotides 865–933 (3’-end of the *NSP* gene), which contained 53 per cent (11/21) of recombination breakpoints, with one present in eight isolates; and the region between nucleotides 1443–1490 (3’-end of the *MP* gene), which contained 24 per cent (5/21) of the recombination breakpoints with one of them present in eight isolates. In PHYVV, two recombination ‘hotspots’ were detected in the DNA-A: regions between nucleotides 341–364 (5’-end of the *CP* gene) and 2471–2543 (intergenic region), each of them containing half of the recombination breakpoints. No recombination ‘hotspots’ were found in DNA-B, where recombination breakpoints were evenly distributed across this genomic segment.

The effect of habitat anthropization in the frequency of recombinants was also analyzed (Table 2). For such analyses, the frequency of recombinant profiles, rather than the frequency of recombinant sequences, was used (see Supplementary Figs S1 and S2). Inter-specific recombinants were more frequent in wild than in let-standing and cultivated habitats (*χ*^2^_1_ = 5.94, *P* = 0.051). Indeed, 83 per cent (4/5) of the inter-specific recombinants came from wild habitats. Frequency of intra-specific recombinants in the PepGMV DNA-A was higher in virus populations from wild than from let-standing and cultivated habitats (*χ*^2^_1_ > 4.67, *P* < 0.010). The level of anthropization of the host population did not affect the frequency of intra-specific recombinants in the DNA-B of PepGMV or in either of the PHYVV genomic DNAs (*χ*^2^_1_ < 2.53, *P* > 0.283).

Thus, the frequency of intra-specific recombinants is high in PepGMV and PHYVV, being higher in the first virus species. In addition, the frequency of PepGMV recombinants decreases in cultivated populations when compared with let-standing and wild ones, but the frequency of PHYVV recombinants is similar across habitats.

### 3.4 Frequency of re-assortants in chiltepin-infecting PepGMV and PHYVV populations

We also analyzed the frequency of PepGMV and PHYVV re-assortants, as re-assortants are also a potential source of genetic diversity for begomoviruses (Briddon et al. 2010). To do so, we followed two approaches: (1) exploring the extent of congruence between the DNA-A and the DNA-B phylogenies and (2) concatenating the DNA-A and DNA-B sequences, and analyzing the presence of recombination breakpoints near the artificial boundaries of both genomic components.

**Figure 3.**
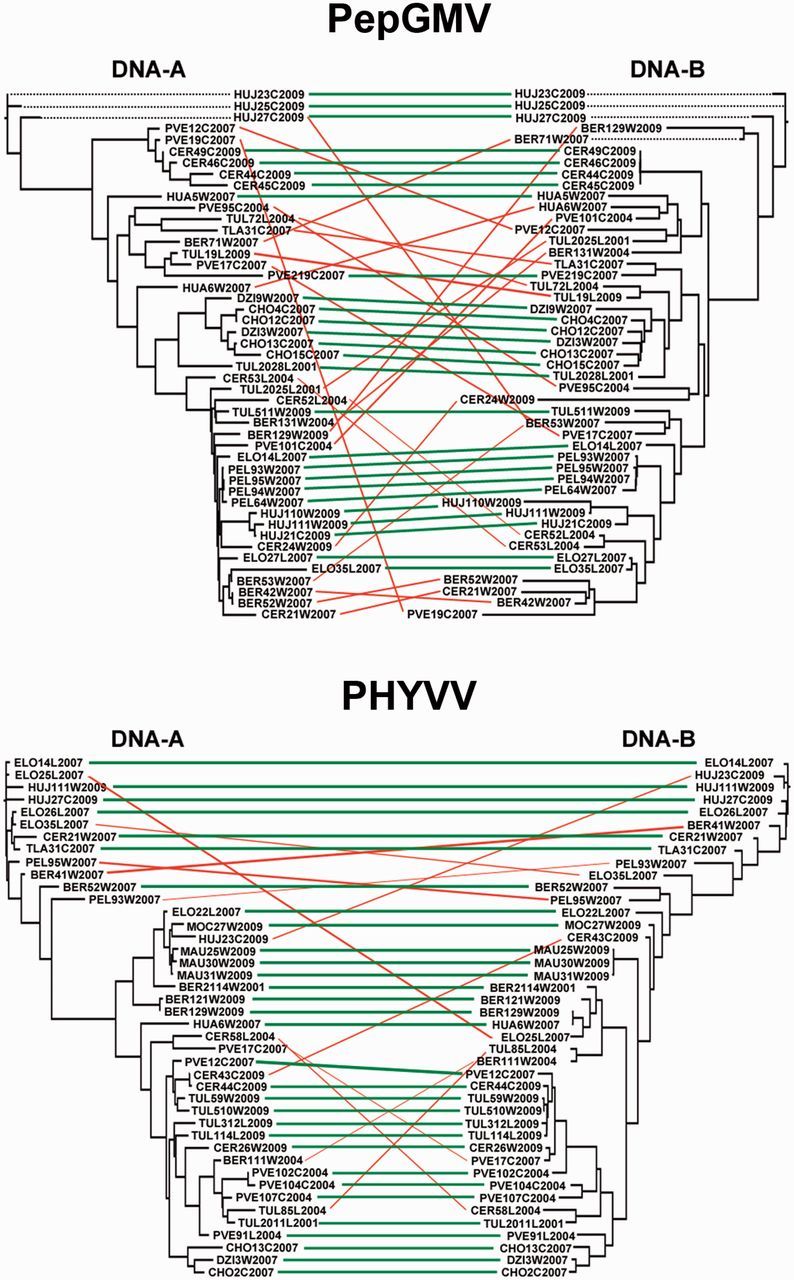
Composite phylogenies of the DNA-A and DNA-B of PepGMV and PHYVV. Lines denote isolates with significant (green) and non-significant (red) phylogenetic congruence between DNA-A and DNA-B as detected by CopyCat. Isolates are identified by a three-letter code indicating the chiltepin population (see Supplementary Table S2) followed by the number of the sample, a one-letter code indicating the level of human management (C, cultivated; L, let-standing; W, wild) and the year of collection.

For PepGMV, the position of 43 per cent (20/47) of the sequences was incongruent between the DNA-A and the DNA-B phylogenies, which was considered as indicative of re-assortants (Fig. 3). The frequency of sequence pairs that showed incongruent phylogenetic positions was higher in wild than in let-standing and cultivated populations (68 per cent, 13/19; 50 per cent, 5/10; and 28 per cent, 5/18, respectively) (*χ*^2^_2_ = 9.36, *P* = 0.009). For PHYVV, 26 per cent (11/42) of the sequences showed incongruent positions between the DNA-A and DNA-B phylogenies, their frequency being similar in the three habitats (21 per cent, 4/19; 30 per cent, 3/10, and 22 per cent, 4/18; in wild, let-standing, and cultivated habitats) (*χ*^2^_2_ = 0.32, *P* = 0.854) (Fig. 3).

Results of the tests for phylogenetic congruence were confirmed by the analysis of the presence of recombination breakpoints in concatenated sequences of the DNA-A and DNA-B. For PepGMV, 53 per cent (25/47) of the sequences had a recombination breakpoint near the boundary of the two genomic DNAs and therefore were considered as re-assortants. These were more frequent in wild than in let-standing and cultivated populations (74 per cent, 14/19; 50 per cent, 5/10; and 33 per cent, 6/18, respectively) (*χ*^2^_2_ = 6.10, *P* = 0.047). For PHYVV, 40 per cent (17/42) of the sequences were re-assortants, their frequency not being affected by the level of anthropization (8/19, 4/11, and 5/12, for wild, let-standing, and cultivated populations, respectively) (*χ*^2^_2_ = 0.11, *P* = 0.949).

These results indicate that re-assortment occurs more frequently in PepGMV than in PHYVV populations. PepGMV re-assortants are more frequent in wild than in let-standing and cultivated habitats, whereas the frequency of PHYVV re-assortants is similar in all habitats.

### 3.5 Association of ecological and epidemiological factors in the genetic diversity of PepGMV populations

**Figure 4.**
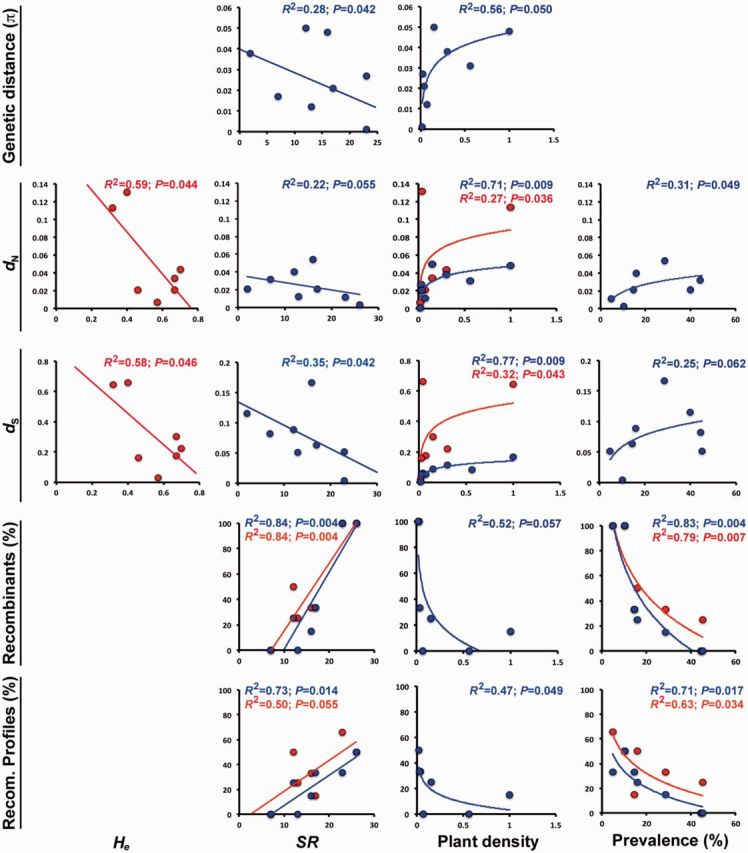
Bivariate relationships between ecological and epidemiological factors of chiltepin populations and evolutionary parameters of PepGMV populations. Significant regressions of plant species richness, chiltepin genetic diversity and plant density, and PepGMV prevalence onto the genetic diversity, the rate of non-synonymous and synonymous substitutions, the frequency of recombinants, and the frequency of different recombinant profiles in PepGMV populations are represented for each genomic component: DNA-A (blue) and DNA-B (red). Species richness (SR) is expressed as number of species; host genetic diversity (*H*_e_) is expressed as expected heterozygosity; host plant density is expressed as plants/m^2^, and virus prevalence as percentage of infected plants over the total of plants sampled. Note the different scales in the *x*- and *y* axis depending on the factor-parameter combination. Data correspond to eight populations with at least four fully sequenced viral isolates.

To test whether changes in habitat species richness, host genetic diversity, host plant density, and virus prevalence were associated with the genetic diversity of PepGMV populations, we calculated *π*, *d*_N_/*d*_S_, *d*_N_, *d*_S__,_ and the frequency of recombinants in the DNA-A and in the DNA-B of isolates from eight PepGMV populations (see Section 2). Using this dataset, we performed bivariate analyses of these evolutionary parameters onto the chiltepin expected heterozygosity (*H*_e_), the habitat species richness (*SR*), the chiltepin plant density, and the PepGMV prevalence in the corresponding host populations considering linear and non-linear models. Results for the best-ranked model explaining each bivariate relationship are shown in Fig. 4. Bivariate analyses for which none of the considered models fitted the data are not shown.

The genetic diversity of the PepGMV DNA-A was negatively associated with *SR* (*R*^2 ^= 0.28; *P* = 0.042) and positively associated with plant density (*R*^2 ^= 0.56; *P* = 0.050). The *d*_N_ and *d*_S_ values were negatively associated with *SR* (*R*^2 ^> 0.22; *P* < 0.055) and positively associated with plant density and PepGMV prevalence (*R*^2 ^> 0.25; *P* > 0.062). Similarly, the percentage of recombinants and of different recombination profiles in the DNA-A was positively associated with *SR* (*R*^2 ^= 0.84; *P* = 0.004 and *R*^2 ^= 0.73; *P* = 0.014, respectively) and negatively associated with plant density and PepGMV prevalence (*R*^2 ^> 0.52; *P* < 0.057 and *R*^2 ^> 0.47; *P* < 0.049) (Fig. 4).

In the DNA-B of PepGMV, genetic diversity and *d*_N_/*d*_S_ was not significantly associated with any of the four variables considered (*r* < 0.24; *P* > 0.263). The *d*_N_ and *d*_S_ values were negatively associated with *H*_e_ (*R*^2 ^> 0.58; *P* > 0.046) and positively associated with plant density (*R*^2 ^> 0.27; *P* > 0.043). The percentage of recombinants, and of different recombination profiles, in the DNA-B was positively associated with *SR* (*R*^2 ^= 0.84; *P* = 0.004 and *R*^2 ^= 0.50; *P* = 0.055, respectively) and negatively correlated with PepGMV prevalence (*R*^2 ^= 0.79; *P* = 0.007 and *R*^2 ^= 0.63; *P* = 0.034) (Fig. 4). No other bivariate analyses yielded a significant correlation, and analyses are not shown. Finally, parallel bivariate analyses for six PHYVV populations yielded non-significant correlations in all cases (*R*^2 ^< 0.28; *P* > 0.097).

Thus, when having an effect, lower chiltepin genetic diversity and habitat species richness increase genetic diversity, *d*_N_ and *d*_S_, and reduce the frequency of recombinants in PepGMV populations; whereas lower plant density and virus prevalence have the opposite effect on the four evolutionary parameters.

## 4 Discussion

Ecosystem biodiversity provides fundamental services that greatly impact human welfare (Naeem et al. 2009). Growing evidence indicates that one such service is the ability to reduce disease risk (Keesing et al. 2010; Ostfeld and Keesing 2012; Joseph et al. 2013). Understanding the mechanisms by which biodiversity loss influences the appearance of disease outbreaks becomes critical to maintain this ecosystem service, even more in the current situation of accelerating biodiversity declines (Mace, Masundire, and Baillie 2005; WWF Living Planet Report 2012). It has been proposed that the reduction of disease risk is achieved by two mechanisms: first, by reducing parasite prevalence/transmission (Keesing, Holt, and Ostfeld 2006, Ostfeld and Keesing 2012) and, as a result of these epidemiological changes, by reducing parasite genetic diversification (Scholle et al. 2013; Lanfear, Kokko, and Eyre-Walker 2014). Although the former mechanism has been studied in a variety of parasites and hosts (Ostfeld and Keesing 2012; Pagán et al. 2012; Laporta et al. 2013; Alexander et al. 2014), the latter has seldom been analyzed (Burdon and Thrall 2008; Scholle et al. 2013; Alexander et al. 2014). Here, we provide evidence of the role that biodiversity has in determining the genetic diversity of two plant begomoviruses—PepGMV and PHYVV—infecting host populations under different levels of anthropization.

The patterns of genetic diversity in chiltepin-infecting PepGMV and PHYVV populations share similarities between them and with those described for other begomoviruses. First, values of genetic diversity are in the same order of magnitude than in other begomoviruses that infect non-cultivated hosts and are generally higher than for crop-infecting begomoviruses (Silva et al. 2011; Lima et al. 2013). Second, PepGMV and PHYVV proteins are under purifying selection, as generally described for most begomoviruses (Sanz et al. 1999; Silva et al. 2011; González-Aguilera et al. 2012; Lima et al. 2013; Rocha et al. 2013). Third, PepGMV and PHYVV populations had relatively high frequency of recombinants and re-assortants, which has been widely documented to have a key role in the evolution of begomoviruses (e.g., Sanz et al. 2000; García-Andrés et al. 2006; Briddon et al. 2010; Martin et al. 2011; Lima et al. 2013; Silva et al. 2014). Fourth, our analysis revealed recombination ‘hotspots’ in the PepGMV and PHYVV genomes already described for other begomoviruses (Fig. 2): A recombination hotspot in the 3’-end of the *CP* gene is present in *Tomato yellow leaf curl virus*, resulting in biologically functional recombinants (García-Andrés et al. 2007). The central region of the *Rep* gene has been also described as highly tolerant to recombination in a number of begomoviruses (Lefeuvre et al. 2007a), and the 5’-end of this gene seems to be a recombination ‘hotspots’ in mono- and bipartite begomoviruses (Lefeuvre et al. 2007a,b).

Although these common features could suggest that both viruses would evolve similarly under conditions of habitat anthropization and biodiversity loss, our results indicate that this is not the case. Habitat anthropization was associated with increased genetic diversity of PepGMV but not of PHYVV. We can only speculate on the causes of this differential effect. For instance, in cultivated chiltepin populations, 80 per cent of plants infected with PHYVV are also infected with PepGMV, whereas this proportions is significantly smaller in let-standing and wild populations (62 per cent and 56 per cent, respectively; *χ*^2^_2_ = 10.10, *P* = 6 × 10^−^^3^) (Rodelo-Urrego et al. 2013). Because co-infection has been shown to reduce virus genetic diversity (Dennehy et al. 2013), it may counteract the effect of reduced biodiversity in PHYVV populations. Alternatively, analyses of association between habitat and PepGMV and PHYVV phylogenies indicated a much stronger association in PepGMV than in PHYVV populations (see MC *P* values in Supplementary Table S4). These analyses suggest that across-habitat migration is higher in PHYVV than in PepGMV populations, which may mask the effects of habitat anthropization. The positive relationship between PepGMV genetic diversity and habitat anthropization corroborates previous data of our group based on analyses of a fragment of the *CP* gene (Rodelo-Urrego et al. 2013). Work of our group has also shown that increased level of anthropization in chiltepin populations is associated with lower biodiversity and higher plant density (Pagán et al. 2012) and with higher PepGMV prevalence (Rodelo-Urrego et al. 2013). Accordingly, here we observed that PepGMV genetic diversity was negatively correlated with habitat species richness and positively correlated with plant density and virus prevalence in chiltepin populations. These results support theoretical predictions on the effect of biodiversity in the genetic diversity of virus populations (Burdon and Thrall 2008; Scholle et al. 2013; Lanfear, Kokko, and Eyre-Walker 2014). Also, our results are compatible with previous reports of faster diversification rates in animal virus populations with higher transmission rates and prevalence (Kurath et al. 2003; Einer-Jensen et al. 2004; Volk et al. 2010). Remarkably, our observations are in apparent contradiction with recent works pointing to a higher genetic diversity of begomovirus populations in non-cultivated than in cultivated hosts (Silva et al. 2011, 2012; Lima et al. 2013; Rocha et al. 2013). However, these works compare the genetic diversity of different viruses, isolated from different habitats. Hence, results derived from such works are difficult to compare with ours as they might be reflecting virus species-specific dynamics, rather than the effect of habitat anthropization. To our knowledge, our work is the first one using an approach that allows a direct association between changes in virus genetic diversity and level of habitat anthropization.

The larger PepGMV genetic diversity at higher levels of habitat anthropization was associated with a small increase of non-synonymous nucleotide substitution rates and with a much larger increase of synonymous substitution rates (Table 1). Accordingly, most sites in the PepGMV genome were under neutral evolution, just a few under purifying selection and none under diversifying selection. Hence, neutral evolution, rather than adaptive selection, might be responsible for the higher PepGMV variability in cultivated chiltepin populations, in agreement with observations in other begomoviruses (Lima et al. 2013). In the absence of adaptive selection, virus populations increase their genetic diversity by accelerating mutation rates or enlarging population sizes (Moya et al. 2000). Analyses of the PepGMV *CP* gene indicated that nucleotide substitution rates did not vary across levels of habitat anthropization (Rodelo-Urrego et al. 2013). In turn, here we showed that increasing host plant density and virus prevalence, conditions that favor higher virus population sizes, were associated with higher *d*_N_ and *d*_S_ values. Since plant density and virus prevalence are higher in cultivated habitats, higher genetic diversity of PepGMV populations in these habitats might be explained by larger virus population sizes.

Because recombination plays a key role in the emergence and evolution of begomoviruses (Lefeuvre and Moriones 2015), we also analyzed the effect of habitat anthropization in the frequency of PepGMV recombinants. This frequency was higher in wild host populations, where species richness is higher, and both chiltepin density and PepGMV prevalence are lower. Such conditions reduce the probability of virus encounter with susceptible individuals, which in turn reduce virus transmission rate and population size, favoring genetic drift. Theory predicts that genetic drift may suffice to promote recombination (Barton 2010), which could explain our results. Indeed, the frequency of PepGMV recombinants was positively correlated with habitat species richness and negatively correlated with plant density and virus prevalence. The effect of habitat anthropization and biodiversity loss in the frequency of PepGMV recombinants is in marked contrast to trends observed for the genetic diversity of PepGMV and suggest that mutation, rather than recombination, accounts for most of the variation in the genetic diversity of PepGMV across habitats. Indeed, such primary role of mutation over recombination has been shown for other begomoviruses (Lima et al. 2013). It could be argued that the frequency of recombinants is not necessarily representative of the contribution of recombination to the genetic diversity of the PepGMV populations when, for instance, lower recombination rates involve: (1) exchange of larger genomic fragments or (2) more genetically divergent parentals. However, the length of the recombinant fragments (DNA-A: 1,000–1,200 nt; DNA-B: 500–700 nt on average), and the genetic diversity between recombination parentals (DNA-A: 0.056–0.057; DNA-B: 0.155–0.169), was similar regardless of the level of habitat anthropization. Therefore, it is likely that our results reflect a smaller contribution of recombination to the genetic diversity of PepGMV populations.

Some cautionary comments, however, are called upon our results. First, we did not attempt to obtain a direct estimate of the relative contribution of mutation and recombination to the genetic diversity PepGMV and PHYVV populations, as it has been done for other begomoviruses (Lima et al. 2013). Hence, our conclusion that mutation accounts for most of the variability in PepGMV genetic diversity across habitats is derived from indirect evidence. Second, the analyses of association between PepGMV evolutionary parameters and ecological/epidemiological factors are based on data from eight chiltepin populations. We are aware that this might be a small sample size. However, it was enough to detect significant, and in many cases strong, correlations between the studied parameters. Third, for these association analyses, estimates of PepGMV evolutionary parameters are based on datasets of four to ten sequences per virus population. This is again a small figure, which may not be representative of the genetic diversity of the virus populations. Nevertheless, rarefaction analyses indicated that four to ten sequences should be enough to capture a significant proportion of the chiltepin-infecting PepGMV genetic diversity (Supplementary Fig. S3). Fourth, it can be argued that rarefaction curves for PHYVV populations are not saturated, which may result in underestimates of PHYVV genetic diversity (Supplementary Fig. S3). However, for the same sample size, our estimates of genetic diversity were still higher in PHYVV than in PepGMV populations. Moreover, the estimates of PHYVV genetic diversity presented here are comparable to those obtained in Rodelo-Urrego et al. (2013) for the same virus populations but using up to seventeen CP sequences per population. Hence, such bias in the estimate of virus genetic diversity should not invalidate our results. Fifth, because virus sequences were sampled in different years, our results may be biased if the virus populations were genetically structured according to sampling date. However, root-to-tip regressions and *N*_ST_ values (calculated as described in Rodelo-Urrego et al. (2013)) indicated that there is no such genetic structure (data available upon request). Analyses in other host-pathogen system would help to tests the generality of our observations.

With the above-mentioned caveats, our results provide evidence of the relevant role that biodiversity and habitat anthropization may have in shaping the genetic diversity of plant virus populations. The reported increase in PepGMV genetic diversity associated with habitat anthropization, and biodiversity loss supports theoretical predictions. Because genetic diversification has been proposed to be involved in the appearance of new viral diseases (Holmes 2009), our results may contribute to understand the factors driving virus emergence. Such factors may have differential effects even in two closely related viruses as PepGMV and PHYVV, which highlights the complexity of developing generally applicable predictive models of virus emergence.

## Data availability

Sequences determined in this work can be found in the EMBL database under accession numbers: LN848751 to LN848928.

## Supplementary data

Supplementary data is available at *VEVOLU* online.

Supplementary Table S1
