# Supplementary material for: The effect of ecosystem biodiversity on virus genetic diversity depends on virus species: A study of chiltepin-infecting begomoviruses in Mexico
Source: Virus Evol. 2015 Jun 1;1(1):vev004. doi: 10.1093/ve/vev004 (PMC5014474; doi:10.1093/ve/vev004)
Supplement: Supplementary Table S1 [file 85f8cb3ae6927c00a299595e89510e9f_Rodelo-Urregoetal_SupplementaryMaterial_Rev.pdf]

Table S1. Primers used for full-length sequencing of the two genomic segments (DNA-A and DNA-B) of PepGMV and PHYVV.

Virus		Primer name	Primer Sequence 5´→3´	Position	T_m (ºC)
PepGMV	DNA-A[1]	PepGMVA2R	CGA TCV CGH AGY TCG TTC TTC	697-717	58.3
		PepGMVA1R	GTT CCA AAT CCC CTA TTT TTC	1,272-1,293	48.5
		PepGMVA1F	CTC CCA RAT AAA AAC GSM ATT C	1,294-1,315	53.0
		PepGMVA3R	GCT CAA TTC GTC ATC CTC CAC	1,473-1,493	54.4
		PepGMVA2F	GCA TTT ACN GCG TTR TRR TAG ACG	2,074-2,097	59.1
PepGMV	DNA-B[2]	PepGMVB2R	CAT CCT TCR ATR TCT ACC ATG AC	964-986	55.3
		PepGMVB1R	TGG ACT TYW CMC ATG TGG ACT A	1,561-1,582	54.8
		PepGMVB1F	CAT CGT TAT GGG WGA AYT GTT T	1,583-1,604	51.1
		PepGMVB3R	GTC ATT CTT TTC RCT VAA RGA YCC	1,746-1,766	57.4
		PepGMVB2F	GAA GTG TTT YGC GAA TAA GAG G	2,121-2,142	53.0
PHYVV	DNA-A[3]	PHYVVA2F	CCG CMA AGA TTA GCC GAA CTG GC	270-292	60.6
		PHYVVA2R	CGC CAT CTA CGT CAC CAT CG	1,505-1,524	55.9
		PHYVVA3F	GAA ATG GAG GAA CCC ANG GTT CC	1,961-1,983	58.8
		PHYVVA1R	TAG AGG AGG ACA GCA GTC TGC	2,125-2,145	56.3
		PHYVVA1F	GCA GAT CTS CCG TCT ATT TGG	2,146-2,166	54.4
PHYVV	DNA-B[4]	PHYVVB2R	CAC ATG NCG WAT RTA RAA MCG TTC C	953-977	59.3
		PHYVVB1R	CTG ACA ACC AAG GAC ATA GC	1,478-1,497	51.8
		PHYVVB1F	CAT CTG TGT GAG TGG TCC CTA TGG	1,498-1,521	59.1
		PHYVVB3R	GCG TCM TTC TTY TCC YTG AAG G	1,822-1,843	58.6
		PHYVVB2F	GCT AYA CCT AAC AAT GWT AAT GG	2,217-2,239	51.7

[1] Position numbered as in sequence of DNA-A from PepGMV-Mo (Acc. No. AY928512).

[2] Position numbered as in sequence of DNA-B from PepGMV-Mo (Acc. No. AY928513).

[3] Position numbered as in sequence of DNA-A from PHYVV (Acc. No. NC_001359).

[4] Position numbered as in sequence of DNA-B from PHYVV (Acc. No. NC_001369).

Table S2. Summary of geographical origin and year of sampling of the PepGMV and PHYVV sequences used in this work.

Virus	Isolate	Population	Habitat	Year
**PepGMV**	BER131W2004	Bernal	Wild	2004
	BER42W2007	Bernal	Wild	2007
	BER52W2007	Bernal	Wild	2007
	BER53W2007	Bernal	Wild	2007
	BER71W2007	Bernal	Wild	2007
	BER129W2009	Bernal	Wild	2009
	CER21W2007	Cerritos	Wild	2007
	CER24W2009	Cerritos	Wild	2009
	DZI03W2007	Dzibilchaltun	Wild	2007
	DZI09W2007	Dzibilchaltun	Wild	2007
	HUA05W2007	Huatulco	Wild	2007
	HUA06W2007	Huatulco	Wild	2007
	HUJ110W2009	El Huajote	Wild	2009
	HUJ111W2009	El Huajote	Wild	2009
	PEL64W2007	Puente Elota	Wild	2007
	PEL93W2007	Puente Elota	Wild	2007
	PEL94W2007	Puente Elota	Wild	2007
	PEL95W2007	Puente Elota	Wild	2007
	TUL511W2009	Tlacuapa	Wild	2009
	CER52L2004	Cerritos	Let-standing	2004
	CER53L2004	Cerritos	Let-standing	2004
	ELO14L2007	Elota	Let-standing	2007
	ELO27L2007	Elota	Let-standing	2007
	ELO35L2007	Elota	Let-standing	2007
	PVE95L2004	Puerto Verde	Let-standing	2004
	TUL2025L2001	Tula	Let-standing	2001
	TUL2028L2001	Tula	Let-standing	2001
	TUL72L2004	Tula	Let-standing	2004
	TUL19L2009	Tula	Let-standing	2009
	CER44C2009	Cerritos	Cultivated	2009
	CER45C2009	Cerritos	Cultivated	2009
	CER46C2009	Cerritos	Cultivated	2009
	CER49C2009	Cerritos	Cultivated	2009
	CHO04C2007	Cholul	Cultivated	2007
	CHO12C2007	Cholul	Cultivated	2007
	CHO13C2007	Cholul	Cultivated	2007
	CHO15C2007	Cholul	Cultivated	2007
	HUJ21C2009	El Huajote	Cultivated	2009
	HUJ23C2009	El Huajote	Cultivated	2009
	HUJ25C2009	El Huajote	Cultivated	2009
	HUJ27C2009	El Huajote	Cultivated	2009
	PVE101C2004	Puerto Verde	Cultivated	2004
	PVE12C2007	Puerto Verde	Cultivated	2007
	PVE17C2007	Puerto Verde	Cultivated	2007
	PVE19C2007	Puerto Verde	Cultivated	2007
	PVE219C2007	Puerto Verde	Cultivated	2007
	TLA31C2007	Tlacuapa	Cultivated	2007

**PHYVV**	BER2114W2001	Bernal	Wild	2001
	BER111W2004	Bernal	Wild	2004
	BER41W2007	Bernal	Wild	2007
	BER52W2007	Bernal	Wild	2007
	BER1210W2009	Bernal	Wild	2009
	BER129W2009	Bernal	Wild	2009
	CER21W2007	Cerritos	Wild	2007
	CER26W2009	Cerritos	Wild	2009
	DZI03W2007	Dzibilchaltun	Wild	2007
	HUA06W2007	Huatulco	Wild	2007
	HUJ111W2009	El Huajote	Wild	2009
	MAU25W2009	Los Mautos	Wild	2009
	MAU30W2009	Los Mautos	Wild	2009
	MAU31W2009	Los Mautos	Wild	2009
	MOC27W2009	Moctezuma	Wild	2009
	PEL93W2007	Puente Elota	Wild	2007
	PEL95W2007	Puente Elota	Wild	2007
	TUL510W2009	Tula	Wild	2009
	TUL59W2009	Tula	Wild	2009
	CER58L2004	Cerritos	Let-standing	2004
	ELO35L2004	Elota	Let-standing	2004
	ELO14L2007	Elota	Let-standing	2007
	ELO22L2007	Elota	Let-standing	2007
	ELO25L2007	Elota	Let-standing	2007
	ELO26L2007	Elota	Let-standing	2007
	PVE91L2004	Puerto Verde	Let-standing	2007
	TUL2011L2001	Tula	Let-standing	2001
	TUL85L2004	Tula	Let-standing	2004
	TUL114L2009	Tula	Let-standing	2009
	TUL312L2009	Tula	Let-standing	2009
	CER43C2009	Cerritos	Cultivated	2009
	CER44C2009	Cerritos	Cultivated	2009
	CHO02C2007	Cholul	Cultivated	2007
	CHO13C2007	Cholul	Cultivated	2007
	HUJ23C2009	El Huajote	Cultivated	2009
	HUJ27C2009	El Huajote	Cultivated	2009
	PVE102C2004	Puerto Verde	Cultivated	2004
	PVE104C2004	Puerto Verde	Cultivated	2004
	PVE107C2004	Puerto Verde	Cultivated	2004
	PVE12C2007	Puerto Verde	Cultivated	2007
	PVE17C2007	Puerto Verde	Cultivated	2007
	TLA31C2007	Tlacuapa	Cultivated	2007

Table S3. Genetic diversity (π), d_N/d_S, d_N and d_S values for each gene in the PepGMV and PHYVV genomes.

	Habitat[1]	PepGMV						PHYVV					
		**CP[2]**	**REn[2]**	**TrAP[2]**	**Rep[2]**	**MP[2]**	**NSP[2]**	**CP[2]**	**REn[2]**	**TrAP[2]**	**Rep[2]**	**MP[2]**	**NSP[2]**
π^3													
	Wild	0.023±0.003	0.022±0.003	0.026±0.004	0.027±0.003	0.077±0.007	0.109±0.009	0.057±0.012	0.061±0.009	0.072±0.015	0.047±0.005	0.080±0.007	0.162±0.014
	Let-standing	0.036±0.004	0.045±0.009	0.045±0.007	0.044±0.004	0.087±0.007	0.105±0.010	0.055±0.006	0.061±0.010	0.066±0.011	0.047±0.006	0.072±0.012	0.084±0.008
	Cultivated	0.065±0.005	0.071±0.009	0.063±0.008	0.072±0.006	0.089±0.009	0.120±0.012	0.052±0.005	0.057±0.009	0.066±0.014	0.045±0.004	0.176±0.014	0.174±0.016
	**ALL**	**0.050±0.006**	**0.047±0.006**	**0.047±0.006**	**0.050±0.004**	**0.083±0.008**	**0.111±0.009**	**0.056±0.005**	**0.059±0.009**	**0.069±0.009**	**0.046±0.005**	**0.079±0.006**	**0.170±0.014**
$d_N/d_S{}^3$													
	Wild	0.041±0.007	0.366±0.024	0.456±0.043	0.186±0.014	0.100±0.006	0.167±0.015	0.047±0.018	0.296±0.015	0.392±0.014	0.111±0.028	0.050±0.006	0.160±0.006
	Let-standing	0.026±0.005	0.450±0.025	0.555±0.038	0.168±0.010	0.076±0.010	0.112±0.023	0.030±0.007	0.311±0.018	0.416±0.020	0.103±0.012	0.036±0.002	0.181±0.006
	Cultivated	0.021±0.003	0.374±0.014	0.336±0.031	0.174±0.016	0.076±0.009	0.152±0.010	0.038±0.008	0.329±0.043	0.405±0.023	0.113±0.009	0.048±0.002	0.171±0.006
	**ALL**	**0.025±0.002**	**0.363±0.008**	**0.407±0.011**	**0.173±0.004**	**0.087±0.002**	**0.147±0.004**	**0.040±0.005**	**0.307±0.007**	**0.398±0.006**	**0.108±0.006**	**0.047±0.002**	**0.173±0.005**
$d_N{}^3$													
	Wild	0.003±0.001	0.015±0.001	0.020±0.001	0.018±0.001	0.037±0.002	0.048±0.003	0.010±0.001	0.036±0.001	0.048±0.002	0.016±0.001	0.015±0.001	0.063±0.002
	Let-standing	0.004±0.001	0.052±0.007	0.047±0.005	0.020±0.001	0.020±0.002	0.031±0.003	0.006±0.001	0.036±0.002	0.045±0.003	0.016±0.001	0.010±0.001	0.074±0.003
	Cultivated	0.004±0.001	0.030±0.001	0.028±0.001	0.032±0.001	0.022±0.002	0.039±0.002	0.008±0.001	0.035±0.002	0.045±0.003	0.013±0.001	0.016±0.001	0.070±0.004
	**ALL**	**0.270±0.08**	**0.030±0.001**	**0.031±0.001**	**0.026±0.001**	**0.028±0.001**	**0.042±0.001**	**0.008±0.001**	**0.036±0.001**	**0.046±0.001**	**0.016±0.001**	**0.014±0.001**	**0.069±0.001**
$d_S{}^3$													
	Wild	0.086±0.006	0.041±0.003	0.043±0.003	0.096±0.004	0.336±0.016	0.290±0.015	0.210±0.007	0.121±0.004	0.122±0.005	0.147±0.005	0.301±0.010	0.396±0.015
	Let-standing	0.141±0.010	0.081±0.004	0.084±0.008	0.119±0.007	0.258±0.012	0.272±0.016	0.216±0.018	0.117±0.008	0.108±0.008	0.151±0.014	0.288±0.018	0.407±0.019
	Cultivated	0.168±0.006	0.115±0.011	0.085±0.003	0.185±0.006	0.288±0.011	0.257±0.007	0.201±0.013	0.108±0.006	0.111±0.006	0.143±0.009	0.323±0.018	0.406±0.024
	**ALL**	**0.153±0.002**	**0.083±0.002**	**0.075±0.001**	**0.150±0.002**	**0.323±0.005**	**0.286±0.004**	**0.213±0.004**	**0.116±0.002**	**0.115±0.002**	**0.145±0.003**	**0.302±0.004**	**0.402±0.006**

[1] Level of anthropisation (Wild; Let-standing; Cultivated; ALL: Wild + Let-standing + Cultivated).

[2] Genes harbored by the PepGMV and PHYVV genomes. CP: coat protein; REn: replication enhancer protein; Rep: replication-associated protein; TrAP: transcription activation protein; MP: movement protein; NSP: nuclear shuttle protein.

[3] Values are mean ± standard error based on 1000 replicates bootstrap of π, and based on pairwise determination of d_N/d_S, d_N and d_S.

Table S4. Analysis of association between habitat and PepGMV and PHYVV phylogenies. *P*-values for three association indexes are presented: Parsimony Score (PS), Association Index (AI), and Monophyletic Clade Size (MC).

	PepGMV		PHYVV	
Habitat	DNA-A	DNA-B	DNA-A	DNA-B
AI	$1.0x10^{-9}$	$1.0x10^{-9}$	$8.0x10^{-3}$	0.160
PS	$1.9x10^{-4}$	$1.9x10^{-4}$	$6.0x10^{-3}$	0.115
MC (Wild)	$5.0x10^{-4}$	$8.8x10^{-4}$	0.038	0.037
MC (Let-standing)	1.000	0.317	0.424	1.000
MC (Cultivated)	$1.0x10^{-4}$	$6.9x10^{-4}$	0.047	0.487

DNA-A	Isolate	Recombination	Habitat	Recombination pattern	Breakpoints		Parentals	
					Beginning	End	Major	Minor
	BER129W2009	Intraespecific	W		1147	2260	CHO13C2007	CER21W2007
	BER131W2004	Intraespecific	W		923	2066	CHO13C2007	CER21W2007
	CER24W2009	Intraespecific	W		847	2108	CHO13C2007	CER21W2007
	DZI09W2007	Intraespecific	W		2506	44	DZI03W2007	HUA05W2007
	HUA06W2007	Intraespecific	W		567	1517	HUA05W2007	DZI03W2007
	HUJ110W2009	Intraespecific	W		1152	2130	CER21W2007	CHO13C2007
	HUJ111W2009	Intraespecific	W		1152	2130	CER21W2007	CHO13C2007
	PEL64W2007	Intraespecific	W		175	2580	CER21W2007	CHO13C2007
	PEL93W2007	Intraespecific	W		847	2108	CHO13C2007	CER21W2007
	PEL94W2007	Intraespecific	W		847	2108	CHO13C2007	CER21W2007
	PEL95W2007	Intraespecific	W		847	2108	CHO13C2007	CER21W2007
	TUL511W2009	Intraespecific	W		894	2101	CHO13C2007	CER21W2007
	CER52L2004	Intraespecific	LS		1152	2108	CHO13C2007	CER21W2007
	ELO14L2007	Intraespecific	LS		847	2108	CHO13C2007	CER21W2007
	ELO27L2007	Intraespecific	LS		847	2169	CHO13C2007	CER21W2007
	TUL2025L2001	Intraespecific	LS		847	2109	CHO13C2007	CER21W2007
	HUJ21C2009	Intraespecific	C		847	2130	CER21W2007	CHO13C2007
	PVE101C2004	Intraespecific	C		845	2106	CHO13C2007	CER21W2007

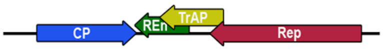

DNA-B	Isolate	Recombination	Habitat	Recombination pattern	Breakpoints		Parentals	
					Beginning	End	Major	Minor
	BER131W2004	Intraespecific	W		905	1455	CER52L2004	CER21W2007
	BER53W2007	Intraespecific	W		908	1476	CER52L2004	CER21W2007
	HUA06W2007	Intraespecific	W		906	1490	CER52L2004	CER21W2007
	PEL64W2007	Intraespecific	W		905	1489	CER52L2004	CER21W2007
	PEL93W2007	Intraespecific	W		905	1489	CER52L2004	CER21W2007
	PEL94W2007	Intraespecific	W		905	1489	CER52L2004	CER21W2007
	PEL95W2007	Intraespecific	W		905	1489	CER52L2004	CER21W2007
	TUL511W2009	Intraespecific	W		892	1489	CER52L2004	CER21W2007
	BER42W2007	*Interspecific*	W		1691	2122	PELO27L2007	PHELO35L2007
	BER52W2007	*Interspecific*	W		965	1415	PBER42W2007	PHELO26L2007
					1646	2108	PBER42W2007	PHELO35L2007
	CER21W2007	*Interspecific*	W		1665	2122	PELO27L2007	PHELO35L2007
	CER24W2009	*Interspecific*	W		673	1325	PHUJ27C2009	PHHUJ23C2009
	HUJ110W2009	*Interspecific*	W		1821	2114	PELO27L2007	PHELO35L2007
	ELO14L2007	Intraespecific	LS		905	1631	CER52L2004	CER21W2007
	ELO27L2007	Intraespecific	LS		906	1490	CER52L2004	CER21W2007
	ELO35L2007	Intraespecific	LS		897	1476	CER52L2004	CER21W2007
	TUL19L2009	Intraespecific	LS		2590	919	CER52L2004	TUL2028L2001
					930	2575	CER52L2004	CER21W2007
	TUL72L2004	Intraespecific	LS		2590	933	CER52L2004	TUL2028L2001
					965	1443	CER52L2004	CER21W2007
	CHO04C2007	Intraespecific	C		905	1489	CER52L2004	CER21W2007
	HUJ21C2009	Intraespecific	C		905	1489	CER52L2004	CER21W2007
	HUJ23C2009	Intraespecific	C		906	1489	CER52L2004	CER21W2007
	HUJ25C2009	Intraespecific	C		906	1489	CER52L2004	CER21W2007
	HUJ27C2009	Intraespecific	C		885	1670	CER52L2004	CER21W2007
	PVE101C2004	Intraespecific	C		906	1490	CER52L2004	CER21W2007
	PVE17C2007	Intraespecific	C		895	1337	CER52L2004	CER21W2007
	PVE19C2007	*Interspecific*	C		1722	2106	PELO27L2007	PHELO35L2007

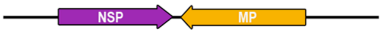

Figure S1. Recombination patterns in the DNA-A and DNA-B of PepGMV isolates. Isolates are presented according to the level of anthropisation of the host population. Recombination patterns are represented by linearizing both genomic DNAs at the nicking site of the invariant nonanucleotide of the origin of replication, and genomic organization of DNA-A and DNA-B is represented accordingly. Colored arrows denote the position and orientation of each gene. CP: Coat protein (blue); REn: Replication Enhancer protein (green); TrAP: Transcriptional Activator Protein (yellow); Rep: Replication protein (red); NSP: Nuclear Shuttle Protein (Purple); MP: Movement Protein (Orange). Data on the type of recombination (intra-or inter-specific), location of recombination breakpoints and parentals is also shown for each recombinant isolate.

DNA-A Isolate	Recombination	Habitat	Recombination pattern	Breakpoints Beginning	Breakpoints End	Parentals Major	Parentals Minor
BER111W2004	Intraespecific	W		2513	341	HUJ23C2009	TUL114L2009
PVE91L2004	Intraespecific	LS		2471	364	HUJ23C2009	TUL114L2009
TUL2011L2001	Intraespecific	LS		2513	341	HUJ23C2009	TUL114L2009
PVE102C2004	Intraespecific	C		2543	341	HUJ23C2009	TUL114L2009
PVE104C2004	Intraespecific	C		2543	341	HUJ23C2009	TUL114L2009
PVE107C2004	Intraespecific	C		2513	341	HUJ23C2009	TUL114L2009

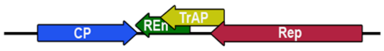

DNA-B Isolate	Recombination	Habitat	Recombination pattern	Breakpoints Beginning	Breakpoints End	Parentals Major	Parentals Minor
BER111W2004	Intraespecific	W		310	1572	PVE91L2004	MOC27W2009
BER41W2007	Intraespecific	W		1091	1458	TLA31C2007	ELO14L2007
PEL93W2007	Intraespecific	W		1603	2211	MAU30W2009	HUJ111W2009
PEL95W2007	Intraespecific	W		606	887	ELO22L2007	ELO14L2007
				1704	2231	ELO22_L_2007	HUJ111W2009
TUL2011L2001	Intraespecific	LS		1973	2233	CER58L2004	HUJ27C2009
TUL85L2004	Intraespecific	LS		310	2007	PVE91L2004	MOC27W2009
HUJ23C2009	Intraespecific	C		1392	1754	ELO14L2007	MOC27W2009
PVE17C2007	Intraespecific	C		176	2560	PVE91L2004	CER44C2009
TLA31C2007	Intraespecific	C		2309	57	BER52W2007	HUJ27C2009

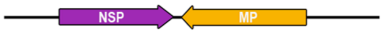

Figure S2. Recombination patterns in the DNA-A and DNA-B of PHYVV isolates. Isolates are presented according to the level of anthropisation of the host population. Recombination patterns are represented by linearizing both genomic DNAs at the nicking site of the invariant nonanucleotide of the origin of replication, and genomic organization of DNA-A and DNA-B is represented accordingly. Colored arrows denote the position and orientation of each gene. CP: Coat protein (blue); REn: Replication Enhancer protein (green); TrAP: Transcriptional Activator Protein (yellow); Rep: Replication protein (red); NSP: Nuclear Shuttle Protein (Purple); MP: Movement Protein (Orange). Data on the type of recombination (intra-or inter-specific), location of recombination breakpoints and parentals is also shown for each recombinant isolate.

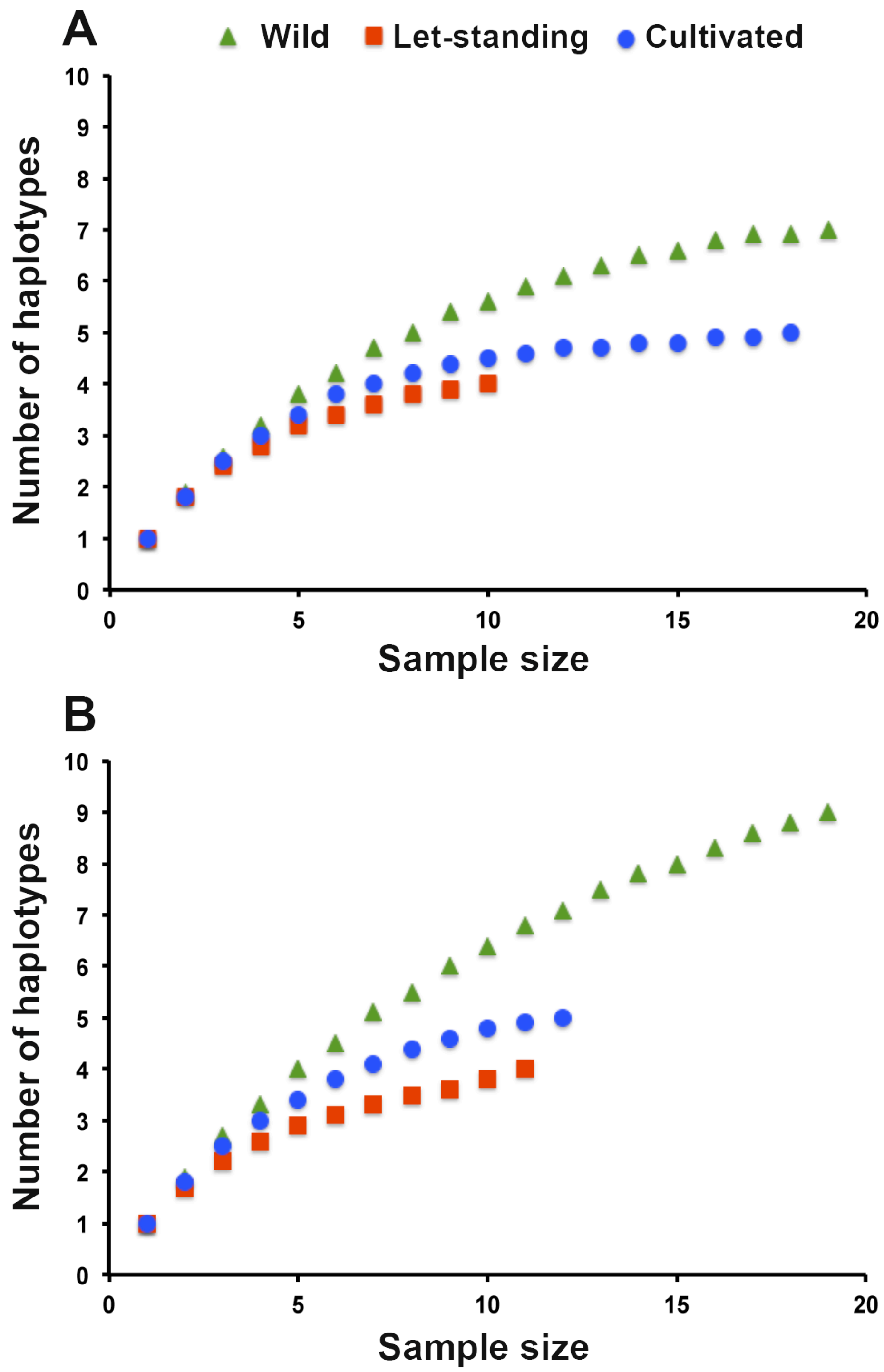

Figure S3. Rarefaction curves for PepGMV (A) and PHYVV (B) populations in each of the three levels of habitat anthropisation considered in this study. Curves were obtained using Analytic Rarefaction 1.3 (http://strata.uga.edu/software/anRareReadme.html).
